# Activation of the Innate Immune Checkpoint CLEC5A on Myeloid Cells in the Absence of Danger Signals Modulates Macrophages' Function but Does Not Trigger the Adaptive T Cell Immune Response

**DOI:** 10.1155/2022/9926305

**Published:** 2022-02-25

**Authors:** Milena J. Tosiek, Kerstin Groesser, Anton Pekcec, Monika Zwirek, Gavuthami Murugesan, Eric Borges

**Affiliations:** ^1^Cancer Immunology & Immune Modulation, Boehringer Ingelheim Pharma GmbH & Co. KG, Birkendorfer Strasse 65, 88397 Biberach an der Riss, Germany; ^2^Research Beyond Borders, Boehringer Ingelheim Pharma GmbH & Co. KG, Birkendorfer Strasse 65, 88397 Biberach an der Riss, Germany; ^3^Medical Research Council (MRC), Protein Phosphorylation and Ubiquitylation Unit, School of Life Sciences, University of Dundee, Dundee DD1 5EH, UK

## Abstract

C-Type lectin receptor 5A (CLEC5A) is a spleen tyrosine kinase- (Syk-) coupled pattern recognition receptor expressed on myeloid cells and involved in the innate immune response to viral and bacterial infections. Activation of the CLEC5A receptor with pathogen-derived antigens leads to a secretion of proinflammatory mediators such as TNF-*α* and IL-6 that may provoke a systemic cytokine storm, and CLEC5A gene polymorphisms are associated with the severity of DV infection. In addition, the CLEC5A receptor was mentioned in the context of noninfectious disorders like chronic obstructive pulmonary disease (COPD) or arthritis. Altogether, CLEC5A may be considered as an innate immune checkpoint capable to amplify proinflammatory signals, and this way contributes to infection or to aseptic inflammation. In this study, we determined CLEC5A receptor expression on different macrophage subsets (in vitro and ex vivo) and the functional consequences of its activation in aseptic conditions. The CLEC5A surface expression appeared the highest on proinflammatory M1 macrophages while intermediate on tumor-associated phenotypes (M2c or TAM). In contrast, the CLEC5A expression on ex vivo-derived alveolar macrophages from healthy donors or macrophages from ovarian cancer patients was hardly detectable. Targeting CLEC5A on noninflammatory macrophages with an agonistic *α*-CLEC5A antibody triggered a release of proinflammatory cytokines, resembling a response to dengue virus, and led to phenotypic changes in myeloid cells that may suggest their reprogramming towards a proinflammatory phenotype, e.g., upregulation of CD80 and downregulation of CD163. Interestingly, the CLEC5A agonist upregulated immune-regulatory molecules like CD206, PD-L1, and cytokines like IL-10, macrophage-derived chemokine (MDC/CCL22), and thymus and activation chemokine (TARC/CCL17) which are associated with an anti-inflammatory or a protumorigenic macrophage phenotype. In the absence of concomitant pathogenic or endogenous danger signals, the CLEC5A receptor activation did not amplify an autologous T cell response, which may represent a protective innate mechanism to avoid an undesirable autoimmune adaptive response.

## 1. Introduction

CLEC5A also known as myeloid DAP12-associating lectin-1 (MDL-1) is a myeloid Syk-coupled pattern recognition receptor which preferentially binds to glycans highly expressed on the surface of pathogens. Upon binding pathogenic antigens, CLEC5A can form multivalent heterocomplexes with other C-type lectins, like DC-SIGN or mannose receptor, but also with Toll-like receptors (TLR). The CLEC5A receptor activation results in the downstream signaling via DAP12 and Syk (as reviewed in [1]).

CLEC5A is mainly expressed on myeloid cells (monocytes, macrophages, neutrophils, and dendritic cells) [2, 3] and can be further upregulated by interferon-*γ* [4]. The key transcription factors reported to control the CLEC5A expression are PU.1, known as a central regulator of myeloid cell differentiation [5] and the nuclear factor erythroid 2-related factor 2 (Nrf2), whose involvement suggests that the CLEC5A expression is regulated by oxidative stress [6].

The ligand for CLEC5A was identified as terminal fucose and mannose moieties of viral glycans, expressed by dengue virus (DV) [7], Japanese encephalitis virus [8] or type A influenza virus [9]. In addition, CLEC5A binds to disaccharides (*N*-acetylglucosamine and *N*-acetylmuramic acid) of bacterial cell walls (e.g., Listeria monocytogenes and Staphylococcus aureus) [1, 10]. Recently, Sung et al. reported that CLEC5A could interact with exosomes released from activated platelets, although the ligand responsible for this interaction has not been identified [11]. Joyce-Shaikh et al. mentioned galectin-9 (Gal9) as a sterile ligand for CLEC5A and proposed a model of Gal9-mediated interaction between CLEC5A-expressing myeloid cells and Gal9-binding T cells [12].

Functionally, the CLEC5A receptor activation triggered by DV or by other pathogens induces the production of proinflammatory cytokines (TNF-*α*, IL-1, IL-6, IL-8, and IL-17A) and chemokines: macrophage inflammatory protein-1 alpha (MIP-1*α*/CCL3), interferon-gamma induced protein 10 kD (IP-10/CXCL10), and macrophage-derived chemokine (MDC/CCL22) [4, 7, 13]. Of note, it was shown that the knockdown of the CLEC5A gene alone was sufficient to suppress the release of proinflammatory cytokines from DV-infected macrophages [7]. In addition to DV infection, a functional blockade of the CLEC5A receptor with different approaches (gene deletion, gene silencing, or *α*-CLEC5A antibody (Ab)) appeared beneficial in some noninfectious conditions, like disease models of chronic obstructive pulmonary disease (COPD) [14] or autoimmune arthritis [4]. These observations suggest that CLEC5A may recognize not only pathogen-associated antigens but also some endogenous danger signals and in consequence could contribute to the pathogenesis of the aseptic inflammation.

In this line, Joyce-Shaikh et al. demonstrated in a model of collagen-induced autoimmune arthritis that activation of the CLEC5A receptor enhanced myeloid cell infiltration and promoted expression of proinflammatory cytokines, e.g., IL-1b, IL-6, IL-17A, and TNF-*α*, leading ultimately to the cartilage damage and bone erosion. Importantly, the presence of LPS strongly enhanced the impact of the *α*-CLEC5A agonistic Ab on TNF-*α* and GM-CSF secretion from bone marrow-derived macrophages. Injections of an agonistic CLEC5A Ab (together with arthritis-inducing Ab mix) in the above model further increased the myeloid cell infiltration into the joint and aggravated disease severity, while the functional blockade of the CLEC5A receptor via gene deletion or injection of CLEC5A-Fc reduced the clinical signs of murine arthritis [4]. Similarly, Wortham et al. observed that CLEC5A expression was upregulated on alveolar macrophages in a mouse model of chronic obstructive pulmonary disease (COPD), as well as in human smokers, possibly because of the oxidative stress triggered by a long-term cigarette smoke exposure. The authors demonstrated, in addition, that CLEC5A activation with an agonistic Ab led to a release of proinflammatory cytokines (TNF-*α* and IL-6) from macrophages and exacerbated lung pathology in mice. Of note, the CLEC5A-mediated cytokine release was significantly enhanced, when the *α*-CLEC5A Ab was accompanied by additional stimuli, like LPS or GM-CSF. Depletion of the CLEC5A gene in mice appeared sufficient to limit the cytokine secretion and to reduce lung pathology when exposed to cigarette smoke [14].

Cheung et al. observed that liver injury triggered in mice with concavalin A resulted in an accumulation of CLEC5A-expressing immature myeloid cells and in consequence led to a lethal shock that resembled systemic inflammatory response syndrome (SIRS) in human. Importantly, a systemic activation of CLEC5A in mice that were not previously exposed to concavalin A-mediated tissue damage did not result in any significant pathology or in systemic cytokine production [3]. Altogether, these findings suggest that in noninfectious conditions, a prior endogenous danger signal, like oxidative stress or tissue damage, may be necessary to sensitize mice to a CLEC5A-mediated immune response.

In this regard, understanding the CLEC5A receptor biology beyond the context of infection, e.g., in aseptic conditions without concomitant activation of pathogen-related receptors, like TLR, may provide new therapeutic opportunities for diseases with the active involvement of CLEC5A-expressing cells like monocytes, macrophages, and neutrophils. In this article, we demonstrate transcriptomic, phenotypic, and functional consequences of the selective activation of CLEC5A-expressing monocyte-derived macrophages (MdM) in aseptic conditions as well as their impact on the activation of autologous T cells. In order to avoid undesirable interactions with other signaling pathways, such as TLR, we selected a CLEC5A-specific monoclonal Ab to study the CLEC5A-mediated immune response in human myeloid cells in aseptic conditions.

## 2. Materials and Methods

### 2.1. Cell Lines

An U937 human myeloid cell line was purchased from American Type Culture Collection (ATCC, USA), while an MDA-MB-231 human breast adenocarcinoma cell line was from Sigma-Aldrich (USA). Both cell lines were maintained in RPMI 1640 Glutamax medium supplemented with 10% fetal calf serum (FCS), both from Gibco, USA.

### 2.2. Human Samples

Blood samples from healthy donors were obtained from DRK-Blutspendeservice (Ulm, Germany).

Human macrophages isolated from ascites fluid and bronchioalveolar lavage fluid (BAL) were provided by Tissue Solutions, UK. All human samples were collected under the appropriate informed consent.

Ascites fluid samples were isolated from 4 female patients aged between 57 and 75 years and diagnosed with high-grade ovarian cancer. BAL samples were collected from 3 healthy donors who have never smoked, aged between 44 and 59 years. Before experiments, cryopreserved samples were thawed in a 37°C water bath and transferred into 50 mL Falcon tubes containing 10 mL FCS. After washing with cold PBS + 2 mM EDTA (both from Gibco, USA) and centrifugation at 300 g for 10 min, the cells were counted with the Neubauer counting chamber (Sigma-Aldrich) and prepared for FACS staining.

### 2.3. Antibodies

Monoclonal human-reactive mouse IgG2B *α*-CLEC5A Ab clone #283834, used for functional assays, and the corresponding isotype control (ctrl) clone #20116 were purchased from R&D Systems, USA. For FACS staining, a PE-labelled Ab clone #283834 was used together with its corresponding isotype ctrl, both obtained from R&D Systems. Other monoclonal Abs used in the manuscript for FACS staining are as follows: HLA-DR, DP, DQ BB515 clone Tu39, mouse IgG2ak BB515 clone G155-178, CD83 BUV737 clone HB15e, mouse IgG1k BUV737 clone X40, CD80 APC-R700 clone L307.4, mouse IgG1k APC-R700 clone X40, CD86 BV650 clone 2331, mouse IgG1k BV650 clone X40, CD163 Alexa647 clone GHI/61, mouse IgG1k Alexa647 clone MOP-C21, CD206 BUV395 clone 19.2, mouse IgG1k BUV395 clone X40, CD14 PE clone M5E2, mouse IgG2ak PE clone G155-178, CD274 (PDL1) BB515 clone MIH1, mouse IgG1k BB515 clone X40, TREM1 BV421 clone 6B1, mouse IgG1k BV421 clone X40, CD14 APC-H7 clone M*φ*P9, CD16 BUV395 clone 3G8, CD64 PerCP-Cy5.5 clone 10.1, CD11c BV650 clone B-ly6, CD4 APC-H7 clone L200, CD4 APC clone RPA-T8, CD8 BUV395 clone RPA-T8, CD8 FITC clone HIT8a, CD56 Alexa 488 clone B159, and CD19 BV605 clone SJ25C1 purchased from BD Biosciences, USA. TREM2 PE clone 237820 and Rat IgG2b PE clone 141945 were purchased from R&D Systems.

### 2.4. Flow Cytometry Analysis (FACS)

Cells were seeded in the 96-well V bottom plates (Greiner Bio-One, Germany) and centrifuged at 300 g for 5 min. Following two washing steps in 150 *μ*L PBS + 2 mM EDTA + 2% FCS (FACS buffer) and centrifugation at 300 g for 5 min, the cells were incubated for 10 min at 4°C in 50 *μ*L FcR blocking reagent (Miltenyi Biotec, Germany) diluted 1 : 100 in FACS buffer. Surface antigens were stained by addition of 50 *μ*L FACS staining solution (FACS buffer containing 2x concentrated FACS antibodies) followed by incubation of the cells for 15 min at 4°C with respective monoclonal antibodies. After two washing steps (as above), flow cytometry analysis was performed at BD FACS Fortessa X20 and analyzed with Flowjo Software Version 10 (both from BD Biosciences, USA).

### 2.5. Generation of U937 CLEC5A KO Cells Using CRISPR/CAS9 Gene Editing

Guide RNAs used for generation of CLEC5A KO were as follows: sgRNA1 (GATCATCTCTGGGCTTATTG, DU60437) and sgRNA2 (GTCATGATGAAGGAGCTGCAG, DU60442) generated by the Division of Signal Transduction and Therapy (DSTT), University of Dundee, UK. Briefly, the U937 cells were transfected with 1 *μ*g of two separate plasmid vectors encoding sense and antisense guide RNAs (p-BABED-Puro-sgRNA1 and pX335-Cas9-D10A-sgRNA2 that target exon 2 of CLEC5A using Lipofectamine™ LTX reagent (Thermo Fisher Scientific, USA). Sixteen hours posttransfection, the U937 cells were selected with 2 *μ*g/mL puromycin (Thermo Fisher Scientific) for 60 h and allowed to recover for a week. Single cells were obtained using FACS sorting and were plated in individual wells of 96-well plates precoated with Growth Factor Reduced Matrigel® (50 *μ*g/mL) (Corning, USA). The viable single cell clones were expanded and screened by genomic DNA-based PCR and DNA sequencing of the targeted locus.

### 2.6. CLEC5A KO Validation with Polymerase Chain Reaction (PCR), DNA sequencing and real-time quantitative PCR (RT-qPCR)

Genomic DNA (gDNA) from U937 WT and U937 CLEC5A KO (here: clone 2.11) was extracted using DNeasy Blood & Tissue Kit (Qiagen, Germany). The PCR amplicon of 596 bp around the target site was PCR amplified using KOD Hot Start DNA Polymerase (Novagen, USA) and CLEC5A forward (5′-AGGGAATGAGTGAAGGAGAGGC-3′) and reverse (5′-AGGGAATGAGTGAAGGAGAGGC-3′) primers. Subsequently, the PCR products were separated on a 1% agarose gel to confirm correct amplicon size, purified with PCR Purification Kit (Qiagen) and ligated into pSC-B-amp/kan vector by using StrataClone Blunt PCR Cloning Kit (both from Agilent Technologies, USA). For each genomic PCR cloning reaction, 20 positive colonies (white colonies in blue-white screening) were amplified, plasmid DNA isolated using QIAprep Spin Miniprep Kit (Qiagen) and Sanger sequencing performed with M13 forward 5′-GTAAAACGACGGCCAGT-3′ and M13 reverse 5′-AACAGCTATGACCATG-3′ primers. Sequenced amplicons of putative knockout were aligned with exon 2 reference CLEC5A sequence using DNA Dynamo DNA Sequence Analysis Software (BlueTractorSoftware Ltd., UK) and CRISPR-induced indels analyzed.

Total RNA from the U937 cells (5*x*10^5^ cells) were extracted using the RNeasy Mini Kit (Qiagen) according to the manufacturer's protocol. RNA (1 *μ*g) was reverse transcribed using the iScript cDNA synthesis Kit (Bio-Rad, USA) according to the manufacturer's protocol. For RT qPCR, 1 *μ*l of diluted cDNA (1 : 20) was mixed with forward and reverse primers (250 nM final concentration each) and SsoFast EvaGreen Supermix (Bio-Rad, USA) in a 96-well plate and run on a Bio-Rad CFX96.

Primer sequences are as follows: CLEC5A forward, TTGTCAACACGCCAGAGAAACTG, and CLEC5A reverse, CAACGCCACCTTTTCTCTTCACG, and *β*-Actin forward, CACCAACTGGGACGACAT, and *β*-Actin reverse, ACAGCCTGGATAGCAACG.

Melting curves were analyzed for purity of the PCR product. Fold changes of CLEC5A transcripts were calculated (2-*ΔΔ*Ct method) and normalized to a housekeeping gene (*β*-actin).

### 2.7. Cytokine Assay with the U937 Cells

The U937 wild-type and U937 CLEC5A KO cells were seeded at 10^5^cells in 200 *μ*L/well in a 96-well flat bottom Nunc Up Cell Plate (Thermo Fisher Scientific) and stimulated for 3 days with 10 nM PMA (Sigma-Aldrich). After stimulation, the cells were washed with medium and transferred into a 96-well high binding plate (Corning, USA), previously coated with 20 *μ*g/mL *α*-CLEC5A Ab or with the corresponding isotype ctrl and incubated at 37°C and 5% CO_2_. After 16 h, 24 h, and 48 h incubation, cell culture supernatants were harvested by centrifugation at 300 g for 5 min and then transferred into 96-well Nunc PP V bottom plates (Thermo Fisher Scientific) and frozen at -20°C for a subsequent cytokine analysis.

### 2.8. Cytokine Measurement in Cell Culture Supernatants

U-Plex 10 Assay Kits for analysis of IL-1b, IL-6, IL-10, IL-12p70, TNF*-α*, VEGFa, IP10, MCP1, TARC, and MDC were purchased from Meso Scale Diagnostics (MSD, USA). The assay was performed as described by the manufacturer. ELISA kits to measure MMP1 (DY901B), MIP1-a (DY270), MIP1-b (DY271), and IL-2 (DY202) were obtained from R&D Systems. The supernatants were tested according to the manufacturer's instructions in 96-well half area plates using 50 *μ*L sample/test.

### 2.9. Isolation of Human PBMC

Peripheral blood mononuclear cells (PBMC) were purified from blood samples as follows. Samples were diluted 1 : 3 with *PBS* + 2 mM EDTA, transferred into 50 mL Leucosep tubes (Greiner Bio-One) containing 15 mL Ficoll-Paque (Cytiva, USA) and centrifuged at 1000 g for 10 min with brakes off. The interphase was carefully collected into 50 mL Falcon tubes. Cell pellets were washed with *PBS* + 2 mM EDTA by centrifugation at 500 g for 10 min followed by two additional centrifugation steps at 200 g for 10 min. After red blood cell lysis with an ACK lysis buffer (Gibco), cells were again washed in *PBS* + 2 mM EDTA, spinned down at 300 g for 5 min, and counted with the COUNTESS II cell counting chamber (Thermo Fisher Scientific).

### 2.10. Isolation of Human Monocytes

Peripheral monocytes were isolated from purified PBMC either with EasySep™ Human Monocyte Enrichment Kit without CD16 Depletion (STEMCELL™ Technologies, Canada) or alternatively with Pan Monocyte Isolation Kit II (Miltenyi Biotec). Monocyte isolation was performed according to the manufacturer's instructions.

### 2.11. Generation of Monocyte-Derived Macrophages (MdM)

#### 2.11.1. M0 (M-CSF) and M2c MdM

Monocytes isolated with the Pan Monocyte Isolation Kit II were seeded in 6-well Nunc Up Cell Plates (Thermo Fisher Scientific) at 2E6 cells in 3 mL/well and differentiated in XVIVO15 medium (Lonza, Switzerland) containing 100 ng/mL M-CSF (R&D Systems) for 9 days. The cells were incubated for an additional of two days either in fresh XVIVO/M-CSF medium (M0 MdM) or in the above medium additionally supplemented with 20 ng/mL IL10 (R&D Systems) (M2c MdM).

#### 2.11.2. GM-CSF MdM

Monocytes enriched and seeded as above were differentiated for 7 days in XVIVO15 medium containing 100 ng/mL GM-CSF (R&D Systems). Next, the cells were stimulated for 24 h in medium supplemented with 20 ng/mL LPS (Sigma-Aldrich) and 50 ng/mL IFNy (R&D Systems).

#### 2.11.3. M1 MdM

Monocytes preenriched from PBMC using EasySep™ Kit were seeded as above and differentiated for 7 days in M1 Macrophage Generation Medium DXF (Promocell, Germany) according to the manufacturer's protocol.

#### 2.11.4. Tumor-Associated Macrophages (TAM), Protocol Based on Benner et al. [15]

Monocytes enriched and seeded as above were differentiated for 9 days in XVIVO15 medium diluted 1 : 2 with tumor-conditioned medium (harvested previously from MDA-MB-231 tumor cell culture) and supplemented with 10% FCS (Gibco), 1 *μ*g/mL IL-4, and 1 *μ*g/mL IL-10 (both from R&D Systems).

### 2.12. Functional Assays with *α*-CLEC5A Ab on MdM

The 96-well high binding plates (Corning) were coated overnight at 4°C with 200 *μ*L PBS solution containing either 10 *μ*g/mL *α*-CLEC5A Ab clone #283834 or its corresponding isotype control clone #20116. Next, the plates were washed twice with cold PBS. MdM were added onto the Ab-/isotype-coated plates in 140 *μ*L of the respective differentiation medium at a concentration of 3.6*x*10^5^ cells/mL and were incubated at 37°C and 5% CO_2_ for 6 h (for transcriptome analysis), 24 h (for cytokine release), or 72 h (for phenotypic FACS analysis).

Cell culture supernatants used for the subsequent cytokine analysis were collected after centrifugation of the plates at 300 g for 5 min and immediately frozen at -20°C. Cells used for the transcriptome study or for phenotypic FACS analysis were prepared as described in the sections Transcriptome Analysis or Flow Cytometry.

### 2.13. Transcriptome analysis (NanoString)

M0 MdM were incubated on culture plates coated either with *α*-CLEC5A Ab or with the isotype ctrl for 6 h as described in the part Functional Assays with *α*-CLEC5A Ab on MdM. Next, the medium was removed, and the cells were carefully washed with PBS. Cell lysates were generated by addition of RLT buffer (Qiagen) at 6500 cells/*μ*L and frozen at -80°C until further analysis.

Human Myeloid-v2 nCounter GX Code Set (NanoString Technologies, USA) was applied for the subsequent transcriptome analysis using 3 *μ*L sample as a template. Data analysis was performed with the nSolver 4.0 software (NanoString Technologies) following the manufacturer's recommendations. First, the gene expression of each sample was normalized to housekeeping genes that met the following criteria: the average transcript count > 25 and the coefficient of variation (CV) < 50%. Furthermore, fold change and false discovery rate (FDR) were calculated for the two groups: *α*-CLEC5A Ab vs isotype ctrl. The final list of curated genes (as represented by the heat map) was generated according to the following criteria: FDR < 0.2, fold change  > 1 and  < −1.5, and transcript counts > 50. The heat map was generated with nSolver 4.0 software (NanoString Technologies).

In case of some manually selected genes of interest, raw counts of samples (calculated with the nSolver software) were in addition graphically visualized as box plots generated with the GraphPad Prism software. The statistical analysis of the above-mentioned samples was performed as described in the section Statistical Analysis.

### 2.14. Autologous MdM+T Cell Coculture Assay

Monocytes were differentiated towards M-CSF (M0) and GM-CSF MdM as described above. Next, 10^5^ macrophages were seeded in the respective differentiation medium containing 25 *μ*g/mL Purified NA/LE Human BD Fc Block (BD Biosciences). Once MdM adhered to the cell culture plates, 10^5^ autologous pan T cells were added. Pan T cells were purified from frozen PBMC with the Pan T Cell Isolation Kit (Miltenyi Biotec) as described by the manufacturer and stimulated in the coculture with macrophages with 0.1 *μ*g/mL *α*-CD3 OKT3 Ab (eBioscience, USA) for 48 h at 37°C. IL-2 release in cell culture supernatants harvested after 48 h was measured by ELISA as described by the manufacturer (R&D Systems). For experiments showing cell proliferation, T cells were first labelled with 1 *μ*M CellTrace™ Violet Cell Proliferation Kit (Thermo Fisher Scientific) according to the manufacturer's protocol and next cocultured with macrophages in the presence of 0.1 *μ*g/mL *α*-CD3 OKT3 Ab. T cell proliferation, defined as CTV dilution in CD4+ and CD8+ lymphocyte populations, was measured by flow cytometry after 5 days of coculture following staining with *α*-CD4 and *α*-CD8 FACS Abs.

### 2.15. Statistical Analysis

Values shown in different graphs are represented as mean ± standard deviation (SD). Statistical analyses (and the graphical representation of the results) were performed with GraphPad Prism 8 (GraphPad Software Inc., USA). Statistical comparison of two different experimental groups in each experiment (Ab vs isotype or WT vs KO) was determined by performing multiple unpaired *t*-tests including the Holm-Sidak method to correct for multiple comparisons. Statistical significance is shown as ^∗^*p* < 0.033, ^∗∗^*p* < 0.002, and ^∗∗∗^*p* < 0.001.

## 3. Results

### 3.1. Monoclonal *α*-CLEC5A Ab Clone # 283834 Selectively Binds CLEC5A and Recapitulates Dengue Virus- (DV-) Mediated Cytokine Response Pattern in the U937 Cells

In order to investigate the role of the CLEC5A receptor in a normal healthy condition, i.e., in the absence of pathogens reported to interact with CLEC5A, we needed to identify a CLEC5A-selective tool, e.g., a monoclonal Ab that exclusively activates CLEC5A in myeloid cells without possible involvement of other surface receptors.

**Figure 1:**
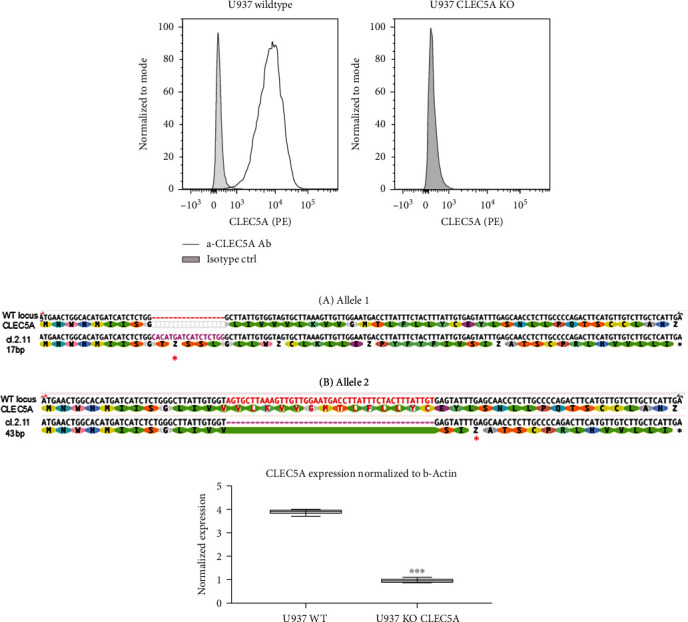
Monoclonal *α*-CLEC5A Ab clone # 283834 binds to U937 wild-type (WT) but not to CLEC5A-deficient (CLEC5A KO) U937 cells. (a) WT (A) or CLEC5A KO (B) U937 cell line was first differentiated with 10 nM PMA for 3 days and then incubated 15 min either with a PE-labelled *α*-CLEC5A Ab or with its corresponding isotype ctrl. *α*-CLEC5A Ab (grey line) vs isotype (grey shadow) binding in FACS is shown as a histogram overlay. (b) CRISPR/Cas9-mediated knockout of CLEC5A at exon 2 in U937 cells was confirmed by sequencing of genomic DNA around the targeted locus. Alignment of CLEC5A WT locus and clone 2.11 CLEC5A KO shows a 17 bp insertion (a, allele 1) and 43 bp deletion (b, allele 2) leading in both cases to a premature stop codon (^∗^) leading to translation termination. (c) The U937 WT and CLEC5A KO cells were differentiated with PMA as described above. RNA from the differentiated cells was extracted, reverse transcribed, and screened for mRNA levels by real-time qPCR for CLEC5A and *β*-actin (housekeeping gene) as a reference. Values represent the *mean* ± *SD* from three technical replicates; ^∗∗∗^*p* < 0.001.

The specificity of the Ab binding to the CLEC5A receptor was tested on a human myeloid cell line, U937 which naturally expresses high levels of CLEC5A mRNA [5]. Among few commercially available human-reactive *α*-CLEC5A Abs, we identified the monoclonal mouse IgG2B clone # 283834 Ab as a selective CLEC5A receptor binder. This Ab demonstrated in flow cytometry a clear binding to the CLEC5A-expressing U937 wild-type but not to the U937 cell line depleted previously from the CLEC5A gene by a CRISPR/Cas9 approach [16] (U937 CLEC5A KO) (Figure 1(a)). The efficiency of the CLEC5A gene depletion in the U937 KO cell line was confirmed by gene sequencing and RT-qPCR techniques (Figures 1(b) and 1(c)).

**Figure 2:**
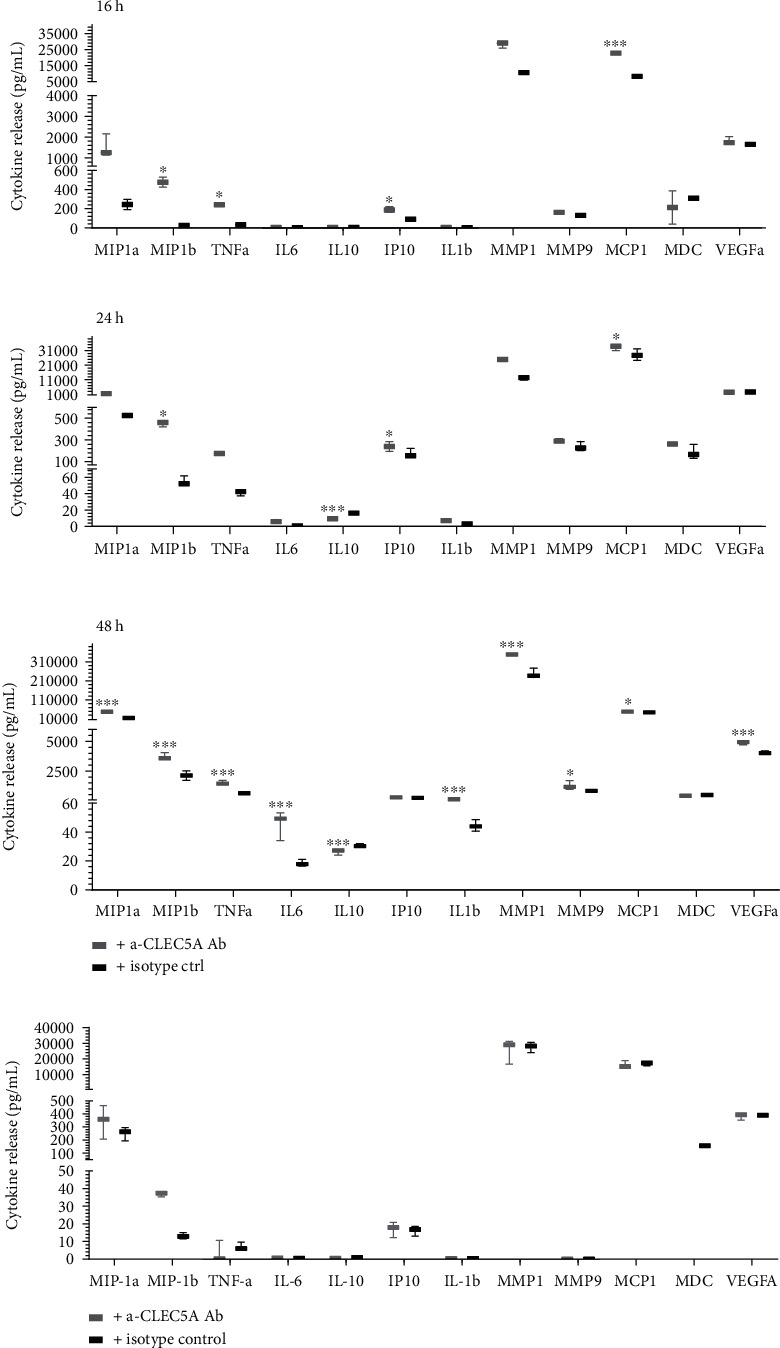
Monoclonal *α*-CLEC5A Ab triggers a time-dependent cytokine secretion from U937 WT but not from U937 CLEC5A KO cell line. (a) PMA-differentiated WT U937 cells were transferred on culture plates previously coated with 20 *μ*g/mL of *α*-CLEC5A Ab (grey symbols) or isotype ctrl (black symbols). Cell culture supernatants harvested after 16 h (upper panel), 24 h (middle panel), and 48 h (bottom panel) were tested for the indicated cytokines with MSD or ELISA kits. (b) PMA-differentiated U937 KO cells were incubated 48 h on culture plates previously coated with 20 *μ*g/mL of *α*-CLEC5A Ab (grey symbols) or isotype ctrl (black symbols). Cell culture supernatants were tested for different cytokines with MSD or ELISA kits. Values represent the mean and SD calculated from three technical replicates/condition; ^∗^*p* < 0.033, ^∗∗^*p* < 0.002, and ^∗∗∗^*p* < 0.001.

Upon engagement of the CLEC5A receptor, the Ab clone #283834 induced in WT U937 cells a time-dependent secretion of different cytokines as compared to the corresponding isotype ctrl (mouse IgG2B). The most pronounced effect of the *α*-CLEC5A Ab was observed after 48 h, resulting in a significant increase in MIP-1a, MIP-1b, TNF-*α*, IL-6, IL-10, and IL-1b. These inflammatory cytokines have been previously reported to be upregulated during infection, for instance, upon CLEC5A-mediated interaction with DV [7]. In addition, the Ab clone # 283834 led to a significant increase of some other soluble inflammatory mediators like metalloproteinases (MMP1 and MMP9), growth factors: vascular endothelial growth factor (VEGFa), and chemokines: monocyte chemoattractant protein-1 (MCP-1/CCL2) (Figure 2(a)).

Importantly, the significant increase in the cytokine secretion as compared to the isotype ctrl was observed only in case the *α*-CLEC5A Ab was incubated with the CLEC5A-expressing WT U937 cells but not with CLEC5A KO U937 cells (Figure 2(b)). Noteworthy, the cytokine response pattern from CLEC5A receptor agonist treated U937 cells was largely comparable to that of the U937 cells exposed to the inactivated DV lysates (Suppl. Fig. 1).

In summary, the Ab clone #283834 selectively binds CLEC5A and induces phenotypically comparable responses to DV, a known CLEC5A receptor agonist. The Ab clone #283834 thus qualifies as a selective CLEC5A receptor agonistic tool to interrogate expression and function of CLEC5A in myeloid cell subsets in the absence of pathogens.

### 3.2. Human Peripheral Myeloid Cells Express CLEC5A

CLEC5A was reported to be expressed by myeloid cells (monocytes, macrophages, dendritic cells, and macrophages) [2], while less is known about the CLEC5A receptor expression in T cells. Here, we measured the CLEC5A surface expression on selected myeloid and T cell populations by FACS using a PE-labelled *α*-CLEC5A Ab clone #283834. Human peripheral myeloid cell populations comprising classical monocytes (CD14+CD16-), nonclassical monocytes (CD16+CD14-/low), and classical dendritic cells (CD11c+) stained positive for CLEC5A. Of note, a clear CLEC5A expression (shown as a single peak) was observed in the majority of CD14+ monocytes (69.2 ± 4.9%). In contrast, CD16+ monocytes and CD11c+ DC had a two-peak pattern of the CLEC5A expression, which marked the presence of both CLEC5A-positive and CLEC5A-negative subpopulations within these cell types. Here, the CLEC5A expression was observed in a minority of CD16+ cells (19.6 ± 7.7%) and in nearly a half of CD11c+ DC (43.6 ± 3.5%).

**Figure 3:**
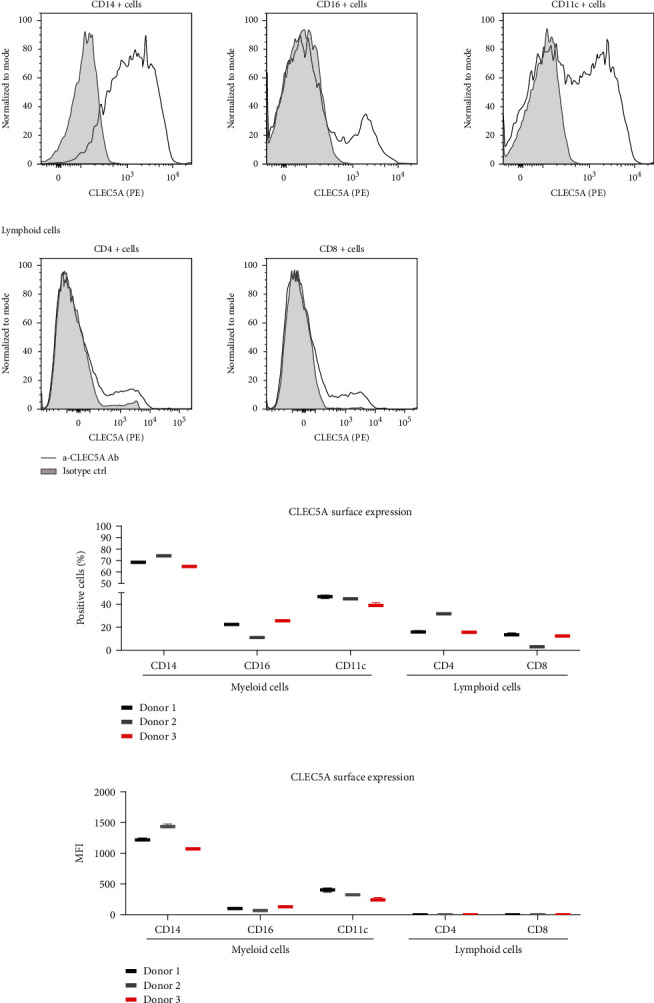
CLEC5A receptor is highly expressed on human myeloid cells. PBMC isolated from healthy donors were stained at 5*x*10^5^ cells in 100 *μ*L/well with a FACS staining solution containing selected antibodies for myeloid and lymphoid cell surface markers: CLEC5A (1 : 40), CD14 (1 : 80), CD16 (1 : 10), CD11c (1 : 20), CD4 (1 : 160), and CD8 (1 : 20). CLEC5A expression was measured by flow cytometry. (a) CLEC5A expression (grey line) vs negative ctrl (grey shadow) on selected immune cell populations: myeloid cells (upper panel) and lymphoid cells (lower panel). Shown are exemplary histograms from one representative donor. (b) Frequency of CLEC5A-expressing cells (shown as percentage of CLEC5A-positive cells) in selected immune cell populations from 3 individual donors. (c) Median fluorescence intensity (MFI) of CLEC5A expression in selected immune cell populations from 3 individual donors. Shown is one representative experiment from two independent measurements (six donors, three technical replicates per donor) with similar outcome.

In contrast to myeloid cells, the majority of CD4+ (78.8%) and CD8+ (90.2%) T lymphocytes did not express CLEC5A on the cell surface (Figures 3(a)–3(c)). This finding confirms the previous observations that the expression of the CLEC5A receptor is mostly restricted to human myeloid cells.

### 3.3. CLEC5A Surface Protein Expression Is Elevated on Proinflammatory M1 Monocyte-Derived Macrophages while Hardly Detectable on Tumor Macrophages

**Figure 4:**
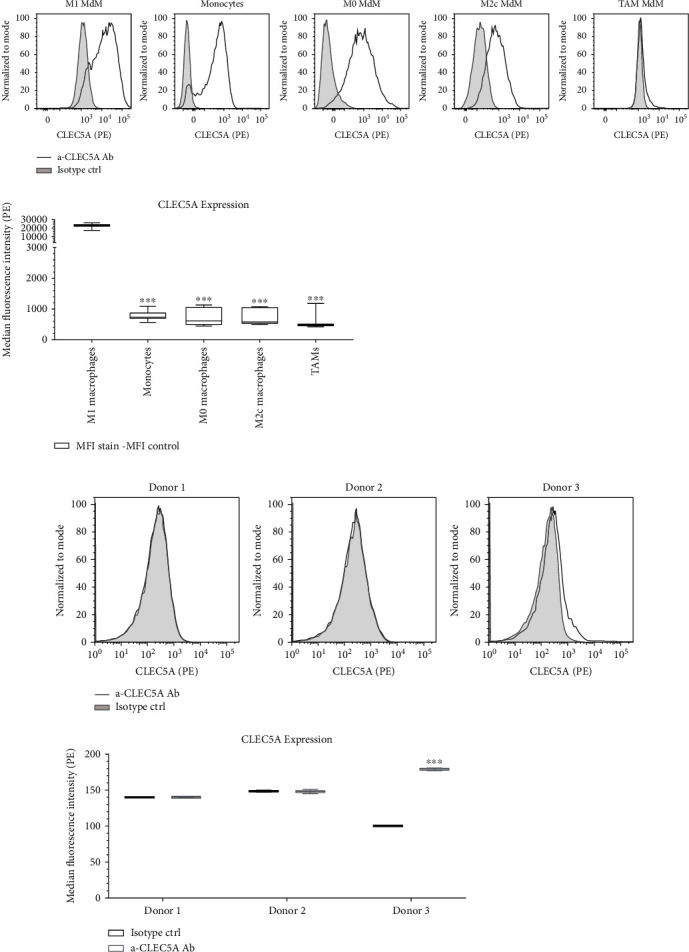
CLEC5A expression is elevated on proinflammatory M1 MdM while minimal on tumor-associated macrophages. (a) Human monocytes from 3 healthy donors were terminally differentiated into several subsets of monocyte-derived macrophages (MdM): M1, M0, M2c, and TAM. 5*x*10^4^ cells in 100 *μ*L/well were FACS stained with a PE-labelled *α*-CLEC5A Ab (1 : 40, grey line) or with isotype ctrl (1 : 40, grey shadow). Histograms show representative stainings from one of three donors per condition. (b) Macrophages isolated from the ascites fluid collected from three patients with advanced ovarian carcinoma were FACS stained at 10^5^ cells in 100 *μ*L/well with a PE-labelled *α*-CLEC5A Ab (1 : 40, grey line) or with isotype ctrl (1 : 40, grey shadow). Data represent three individual donors tested in technical duplicates. The CLEC5A expression is shown as an antibody vs isotype histogram overlay (upper panel) or as median fluorescence intensity (MFI) of the CLEC5A Ab staining or isotype ctrl for each group tested (lower panel). Values represent the mean and SD of each donor; ^∗^*p* < 0.033, ^∗∗^*p* < 0.002, and ^∗∗∗^*p* < 0.001.

We aimed to understand how the CLEC5A receptor expression may be modulated in response to different (e.g., proinflammatory vs protumorigenic) stimuli that drive monocytes towards several MdM subsets. To this end, we isolated monocytes from human peripheral blood and differentiated them *in vitro* towards different MdM subpopulations, proinflammatory M1, “neutral” M0, and protumorigenic M2c (reviewed in [17, 18]), and recently described *in vitro* tumor-associated macrophages (TAM) [15]. Next, we stained the CLEC5A surface protein expression in the above listed subpopulations with the Ab clone #283834 vs corresponding isotype ctrl (Figure 4(a)). The CLEC5A expression was significantly elevated in proinflammatory M1 MdM as compared to monocytes and to other MdM subsets (M0 and M2c), while monocyte differentiation towards TAM resulted in a reduction of CLEC5A expression. To corroborate more on the hypothesis that macrophages exposed to tumor microenvironment may downregulate the CLEC5A receptor, we isolated macrophages from the ascites fluid of patients diagnosed with ovarian carcinoma and tested them on CLEC5A surface receptor expression by FACS (Figure 4(b)). In line with the findings on *in vitro* differentiated TAMs, the *ex vivo*-derived cancer-related macrophages showed only very low CLEC5A expression. As compared to the isotype ctrl, CLEC5A surface expression was detectable only in one of the three patients tested.

Similarly, only minimal CLEC5A expression could be observed on tissue-derived (alveolar) macrophages isolated from healthy donors (Suppl. Fig. 2). Altogether, this may suggest that the elevated CLEC5A protein expression on myeloid cells is rather associated with a proinflammatory condition in contrast to normal tissue or cancer.

### 3.4. *α*-CLEC5A Agonistic Ab Induces a Mixed Pro- and Anti-Inflammatory Cytokine Response from Human MdM

**Figure 5:**
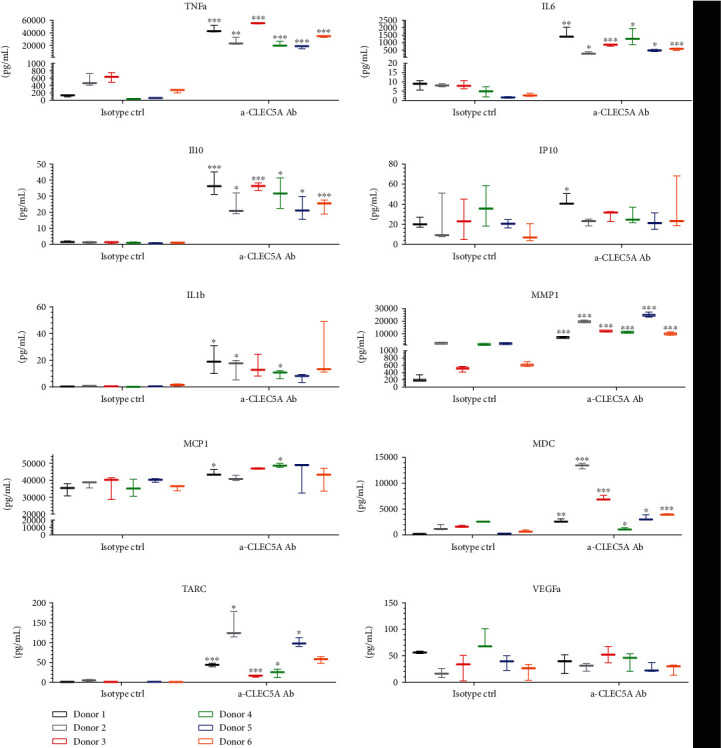
Antibody-mediated activation of the CLEC5A receptor on human MdM leads to a mixed cytokine response. Monocytes isolated from six donors were differentiated into M0 MdM and then transferred onto culture plates coated with 10 *μ*g/ml *α*-CLEC5A Ab or with the isotype ctrl. Cell culture supernatants were harvested 24 h later and tested for several cytokines with MSD or ELISA kits. Values represent the mean ± SD from three technical replicates per each donor. Shown is the summary result of two independent experiments with n = 6 donors; ^∗^*p* < 0.033, ^∗∗^*p* < 0.002, and ^∗∗∗^*p* < 0.001.

In order to understand the functional effects of CLEC5A agonist under noninfectious conditions, we studied the cytokine response in M0 MdM (shown in Figure 4 to express an intermediate CLEC5A expression level) when exposed to the agonistic *α*-CLEC5A Ab. To this end, we generated M0 MdM from peripheral monocytes and incubated them for 24 h either with the Ab clone #283834 or with the corresponding isotype ctrl. In agreement with the data generated earlier on the CLEC5A-expressing U937 cell line (Figure 2), M0 MdM exposed to the *α*-CLEC5A Ab significantly upregulated (in most donors tested) the secretion of cytokines involved in the CLEC5A-mediated response to DV, like TNF-*α*, IL-6, IL-10, and IL-1b (Figure 5). Furthermore, we observed a CLEC5A Ab-mediated release of cytokines reported previously to mark alternatively activated, noninflammatory macrophages [18], like CCL22/MDC (significant upregulation in all donors tested) and CCL17/TARC (statistical significance in 5 out of 6 donors). Altogether, the CLEC5A agonist clone #283834 led to a mixed cytokine response pattern in M0 MdM where the release of classical inflammatory cytokines may be at the same time fine-tuned by anti-inflammatory ones [19].

### 3.5. CLEC5A Agonist Triggers Broad Changes in the Expression of Myeloid Cell-Related Genes

**Figure 6:**
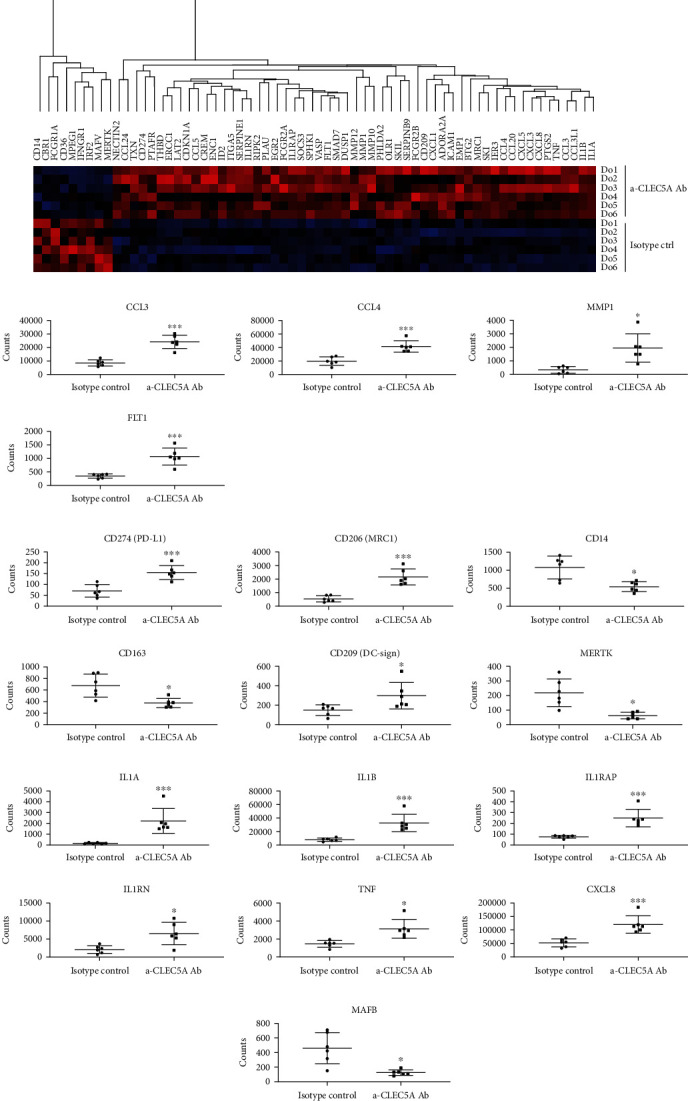
CLEC5A agonistic Ab induces transcriptome changes in myeloid cell-specific genes. M0 MdM generated from same six donors as in Figure 5 were incubated for 6 h either with the *α*-CLEC5A Ab or with the isotype ctrl (10 *μ*g/mL each) and then processed for the transcriptome analysis with NanoString nCounter. (a) Heat map of differentially expressed genes (preselected according to the quality criteria as described in the Materials and Methods) in M0 MdM exposed to the *α*-CLEC5A Ab (upper rows; each donor represented separately) as compared to the isotype ctrl (bottom rows). (b–e) Box plots showing expression of selected genes in M0 MdM upon treatment with *α*-CLEC5A Ab or isotype. Depicted are representative genes encoding soluble mediators (b), myeloid cell-specific surface receptors (c), selected cytokines from the IL-1 cytokine family with their functional partners (d), and a myeloid cell-specific transcription factor MAFB (e). Values represent mean and SD calculated from NanoString raw counts (each dot represents one individual donor). Shown is the summary result from two independent experiments with six donors; ^∗^*p* < 0.033, ^∗∗^*p* < 0.002, and ^∗∗∗^*p* < 0.001.

To understand functional changes driven by the CLEC5A receptor in M0 MdM in more detail, we performed a transcriptome analysis (NanoString nCounter) of M0 MdM generated from the same six healthy donors as above. To this end, terminally differentiated M0 MdM were incubated either with the agonistic *α*-CLEC5A Ab or with the isotype ctrl for 6 h (to track the early transcriptome changes) and were next analyzed for differential gene expression with the nCounter myeloid panel. Data curation performed according to NanoString's quality recommendation resulted in a list of 65 genes that passed the selection criteria (Figure 6(a)). Among the genes that were significantly upregulated in the samples exposed to the *α*-CLEC5A agonistic Ab (as compared to the isotype ctrl), we recognized some genes that encode soluble mediators upregulated on the protein level in the earlier assays, e.g., CCL3 (encoding MIP-1a), CCL4 (MIP-1b), matrix metalloproteinase MMP-1, and FLT1 (VEGFR-1) that encodes a receptor for VEGF (Figure 6(b)). In addition, the CLEC5A receptor agonist induced significant changes in the expression of genes transcribed to myeloid cell-specific surface receptors, for instance, downregulation of CD14 (monocyte differentiation antigen), CD163 (marker of M2 macrophages), and MERTK (MER protooncogene tyrosine kinase). Among genes encoding receptors significantly upregulated by the CLEC5A agonist, we identified MRC-1 (macrophage mannose receptor-1) also known as CD206 antigen, DC-SIGN (dendritic cell-specific intercellular adhesion molecule-3-grabbing non-integrin) also known as CD209, and CD274 that encodes PD-L1, an important biomarker in the context of antitumor immune response [20] (Figure 6(c)).

Moreover, the activation of the CLEC5A receptor with the Ab clone #283834 led to a significant upregulation of genes belonging to the IL-1 cytokine family or its functional partners (Figure 6(d)), e.g., IL-1b and TNF-*α* that in addition were significantly enhanced on the protein level (see Figure 5). The last observation may indicate CLEC5A-mediated inflammasome activation in macrophages, reported previously in the context of DV infection [21] but to our knowledge not in aseptic inflammation.

Furthermore, the CLEC5A activation in M0 macrophages led to a significant downregulation of MAFB (MAF BZIP Transcription Factor B), reported previously to support an anti-inflammatory phenotype of human macrophages [22] (Figure 6(e)).

Our observations on the CLEC5A-driven transcriptomic regulation of the selected key genes that mark a proinflammatory or anti-inflammatory macrophage phenotype were confirmed by a qRT-PCR study which reproduced the statistically significant downregulation of CD163, CD14, and MERTK genes, as well as previously observed downregulation of MAFB and upregulation of FLT1 markers (Suppl. Fig. 3a)). In case of three genes tested (CD206, CD209, and CD274), we noted, however, some discrepancy between the two technical approaches (NanoString vs qRT-PCR), i.e., the significant upregulation of these genes upon treatment with the *α*-CLEC5A Ab as revealed by NanoString, while the downregulation is shown in the qRT-PCR study. This effect was possibly related to the fact that the donors included in NanoString and in qRT-PCR experiments were different (due to technical limitations). Moreover, the number of samples tested by qRT-PCR was inferior to that in the transcriptomics experiment.

In addition to M0 (M-CSF) macrophages, we performed a similar transcriptome analysis in GM-CSF macrophages, since GM-CSF was reported previously to boost the CLEC5A expression in MdM [2]. In contrast to the clear transcriptomic changes observed in M0 macrophages, exposure to the *α*-CLEC5A Ab did not trigger any significant gene expression reprogramming in GM-CSF macrophages (data not shown), while qRT-PCR indicated only two genes significantly modulated by CLEC5A in GM-CSF MdM: upregulation of MAFB and CLEC5A, as shown in the Suppl. Fig. 3B.

### 3.6. CLEC5A Agonist Leads to a Concomitant Regulation of Receptors Involved in T Cell Stimulation as well as Markers of M2-Like/TAM Macrophages

**Figure 7:**
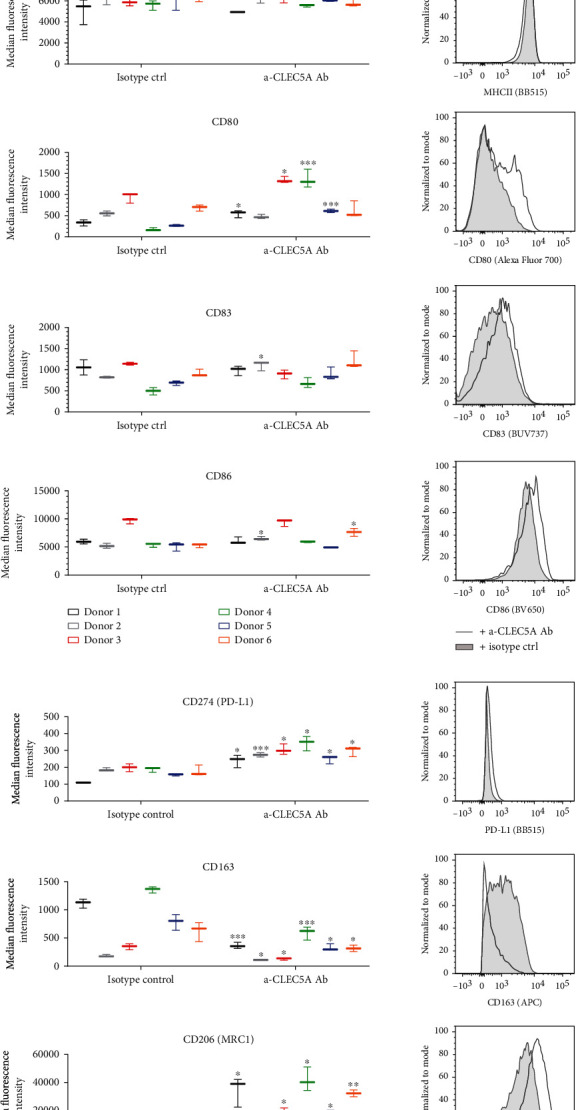
CLEC5A receptor agonist modulates the surface expression of other myeloid cell-specific receptors. M0 MdM generated from same donors as in Figures 5–6 were incubated for 72 h on culture plates coated either with the *α*-CLEC5A Ab or with the isotype ctrl (10 *μ*g/mL) and then FACS stained with directly labelled monoclonal Abs against different membrane receptors. For FACS staining, monoclonal antibodies were diluted 1 : 40 in 100 *μ*L FACS buffer. Graphs depict the expression of each marker shown as MFI for each donor tested, while histograms illustrate an exemplary FACS fluorescence in one representative donor (grey line: MdM incubated with *α*-CLEC5A Ab; grey shadow: MdM incubated with isotype ctrl). Values represent the mean and SD from three technical replicates per each donor. Shown is the summary result from two independent experiments with *n* = 6 donors; ^∗^*p* < 0.033, ^∗∗^*p* < 0.002, and ^∗∗∗^*p* < 0.001.

Our finding from the transcriptome study that the CLEC5A activation can modulate not only cytokines but also the expression of some myeloid cell-specific surface receptors led us to a follow-up FACS characterization of selected myeloid cell receptors on macrophages. Indeed, after a 72 h incubation of M0 MdM with the *α*-CLEC5A Ab, we could confirm our key transcriptomic observations also on the protein level, e.g., CD14 receptor downregulation (significant in 4/6 donors) and PD-L1 receptor upregulation (statistically significant in all donors tested) (Figure 7).

In addition, the CLEC5A receptor agonist enhanced the expression of CD80, a costimulatory receptor involved in T cell activation (effect significant in 4/6 donors), while it had little or no impact on the surface expression of other receptors involved in T cell costimulation or Ag presentation, e.g., CD86, CD83, or MHCII (Figure 7(a)). Regarding myeloid cell receptors reported to orchestrate the inflammatory response, like triggering receptors expressed on myeloid cells: TREM1 (immune amplifier) and TREM2 (negative immune regulator) [23], we observed only limited and variable effect of the *α*-CLEC5A Ab on their surface expression (Suppl. Fig. 4). Importantly, the activation of the CLEC5A receptor with the Ab clone # 283834 had a significant impact on the expression of classical anti-inflammatory M2-like markers like CD163 and CD206 [18]. Interestingly, the expression of these key M2-like markers was regulated by the *α*-CLEC5A Ab in the opposite direction: while all donors significantly downregulated CD163, at the same time they upregulated CD206 protein (Figure 7(b)); the latter observation confirmed the data from the transcriptomic study shown in Figure 6. In line with the concomitant upregulation of PD-L1, this finding may suggest that the CLEC5A activation induces not only proinflammatory markers that could contribute to the immune response during infection but at the same time upregulates receptors that turn MdM towards an anti-inflammatory phenotype and may limit T cell response.

### 3.7. Activation of the CLEC5A Receptor on MdM Does Not Support Autologous T Cell Priming

Having observed transcriptomic and phenotypic changes in MdM exposed to the CLEC5A agonistic Ab, we speculated on potential functional consequences which CLEC5A-activated MdM may exert on surrounding T cells. First, we tested the hypothesis that the *α*-CLEC5A Ab may directly regulate T cell activation. As shown in Figure 3, the vast majority of CD4 and CD8 T lymphocytes do not express CLEC5A, in contrast to myeloid cells. Incubation of purified human T cells in the presence of the *α*-CLEC5A Ab (as compared to the isotype ctrl) did not exert any significant effect on the secretion of IL-2, a T cell-specific cytokine. This observation was true in case of naïve (and memory) T cells (IL-2 under detection level, data not shown) as well as in case of polyclonally activated T cells (Suppl. Fig. 5). To this end, we excluded a hypothesis that the *α*-CLEC5A Ab may impact T cell activation directly.

**Figure 8:**
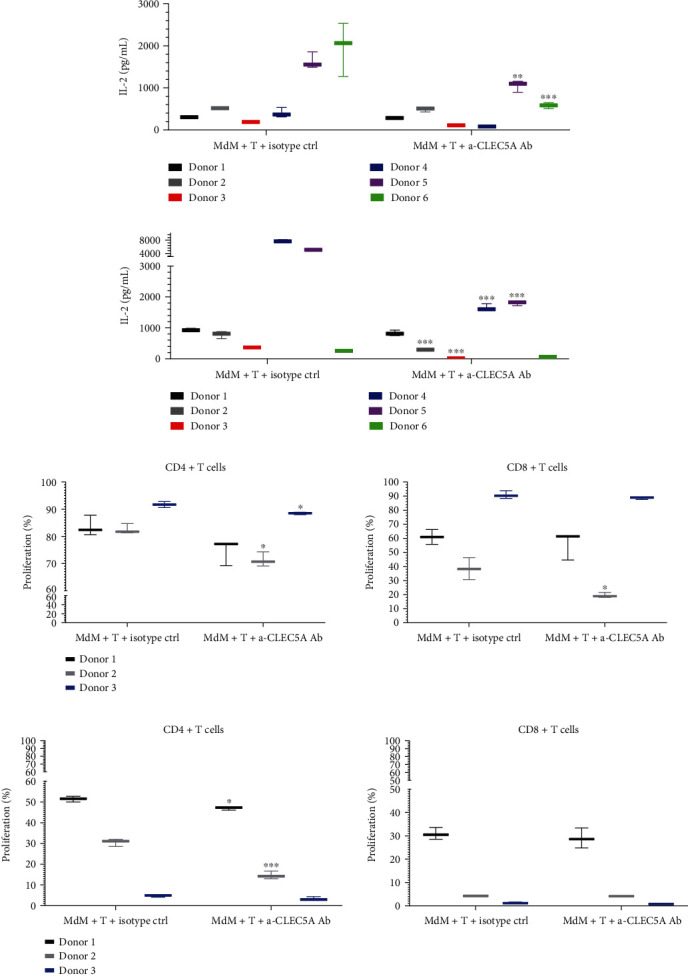
CLEC5A receptor activation on MdM does not support autologous T cell priming. MdM generated from monocytes in the presence of M-CSF (a) or GM-CSF (b) were cocultured for 48 h with pan T cells that were purified from same donors and preactivated with 0.1 *μ*g/mL a-CD3 Ab. T cell-derived IL-2 secretion was measured by ELISA in cell culture supernatants. Proliferation of CD4+ (left) and CD8+ T cells (right) cocultured for 5 days with M-CSF (c) or GM-CSF (d) macrophages in the presence of 0.1 *μ*g/mL *α*-CD3 Ab was measured by flow cytometry. The cells were stained in 100 *μ*L FACS buffer containing 1 : 20 diluted FACS antibodies against CD4 and CD8 antigens. Values represent the mean and SD from three technical replicates per donor. Shown is the summary result of two independent experiments with six donors (a and b) and one experiment with three donors (c and d); ^∗^*p* < 0.033, ^∗∗^*p* < 0.002, and ^∗∗∗^*p* < 0.001.

In order to test a potential indirect contribution of the CLEC5A agonist to T cell activation (via CLEC5A-expressing myeloid cells), we cocultured M0 (M-CSF) MdM with autologous T cells and with a suboptimal T cell activating stimulus (*α*-CD3 Ab) either in the presence of the *α*-CLEC5A Ab or with isotype ctrl. Next, we evaluated T cell activation defined as an increase in IL-2 in the cell culture medium. While MdM seeded alone did not produce any measurable levels of IL-2 (data not shown), T cells incubated together with MdM and with a T cell-activating *α*-CD3 Ab released IL-2, the amount of which was donor-dependent. In four out of six donors tested, the incubation of M0 MdM with the *α*-CLEC5A Ab decreased IL-2 production, as compared to the isotype ctrl. In two of these cases, the difference was statistically significant, suggesting that CLEC5A activation on macrophages generally does not support and in some donors may rather impair the autologous T cell activation (Figure 8(a)). To understand whether a similar impact on the T cell response following CLEC5A activation on macrophages could be achieved in case MdM were differentiated under different conditions, we generated MdM from same donors, this time in the presence of GM-CSF.

While cultivation of GM-CSF MdM with autologous T cells generally led to a higher IL-2 secretion as compared to M-CSF MdM, the addition of the *α*-CLEC5A Ab significantly decreased IL-2 production in four out of six donors tested (Figure 8(b)).

In line with the diminished IL-2 secretion, proliferation of T cells cultured with macrophages exposed to the *α*-CLEC5A Ab was impaired. The presence of the CLEC5A-activated M-CSF MdM slightly, yet statistically significant, inhibited the expansion of CD4+ (helper) T cells in two out of three donors. In case of one donor, the expansion of CD8+ (cytotoxic) T cells decreased significantly (Figure 8(c)).

Similarly, limited proliferation of CD4+ T cells was observed if lymphocytes were cultured in the presence of CLEC5A-activated GM-CSF macrophages (Figure 8(d)).

Altogether, the activation of the CLEC5A receptor on CLEC5A-expressing MdM cocultured with autologous T cells suggested an attenuated T cell response.

Interestingly, when MdM were replaced with supernatants harvested from macrophages previously exposed either to the *α*-CLEC5A Ab or to the isotype ctrl, there was no noticeable effect of CLEC5A on T cell proliferation anymore (Suppl. Fig. 6). Thus, under our experimental conditions, soluble factors released from CLEC5A-activated macrophages were not sufficient to attenuate T cell activation.

## 4. Discussion

It has been previously reported that CLEC5A is not only involved in the host recognition of viral and bacterial pathogens but can also mediate the sensing of endogenous yet unknown ligands during the aseptic inflammation. In the context of infection, the CLEC5A receptor biology has been well described, including associations with other receptors and pathways. In contrast, much less is known about the activation of the CLEC5A receptor without any accompanying exo- or endogenous stimuli and whether such aseptic activation could reprogram human myeloid cells towards their direct or indirect (e.g., via T cell priming) contribution to inflammation.

Hence, in this study we investigated phenotypic and functional consequences of the CLEC5A receptor activation in immune cells under normal noninflammatory conditions. The data presented in this manuscript was generated with a highly specific yet a single clone of *α*-CLEC5A Ab; therefore, we recognize that some observations on the CLEC5A biology may be limited by this approach. We resigned, however from alternative tools to trigger CLEC5A activation, for instance, from deactivated dengue viral particles, because such tools could function as a pathogen-associated molecular pattern (PAMP) and in consequence - activate not only the CLEC5A receptor but also other receptors reported to interact with DV, like DC_SIGN or mannose receptors [24].

First, we studied the CLEC5A surface expression on different immune cell populations *in vitro* and *ex vivo*. Not surprisingly, peripheral myeloid cells expressed high levels of CLEC5A, while its expression on T lymphocytes was hardly detectable. Earlier studies reported that CLEC5A mRNA expression was much higher in GM-CSF-differentiated macrophages as compared to M-CSF-derived ones [2]. In line with this observation, in our hands the CLEC5A protein expression was significantly elevated on proinflammatory M1 GM-CSF MdM as compared to monocytes and to other MdM subsets.

TNF-*α* was recognized by others as the key stimulus that could enhance CLEC5A expression on macrophages (boosted CLEC5A mRNA and protein expression in mouse BM-macrophages in vitro) [4]. In addition, a chronic exposure to cigarette smoke increased the CLEC5A expression on alveolar macrophages in mice and in human as compared to controls [14]. Alveolar macrophages isolated from healthy nonsmokers demonstrated also in our hands only a minimal CLEC5A expression, suggesting that probably an additional, danger-like stimulus is required to induce or to enhance the CLEC5A expression on myeloid cells in a normal nonpathologic condition.

In autoinflammatory disorders, like rheumatoid arthritis (RA) or Adult-onset Still's disease (AOSD), the percentage of CLEC5A-expressing monocytes and their level of CLEC5A expression (MFI) were significantly elevated as compared to healthy controls. Moreover, the CLEC5A expression positively correlated with the parameters of disease activity and with the secretion of proinflammatory cytokines, while it decreased after a therapy [25, 26].

In the context of cancer, the CLEC5A expression on tumor-associated myeloid cells significantly correlated with a decreased overall survival of patients with glioma [27]. Similarly, high CLEC5A expression was proposed as a negative prognostic factor in patients with gastric cancer [28]. Studies on gene expression profiling in ovarian cancer suggested CLEC5A as one of predictive genes for the cancer prognosis [29, 30]. Interestingly, in our study, macrophages isolated from ovarian cancer patients showed only a minimal CLEC5A expression. Nevertheless, a broader study including more patients and different cancer types would be necessary to elaborate on a potential role of CLEC5A (or lack of thereof) in cancer.

Activation of M-CSF-differentiated MdM (M0), showing intermediate CLEC5A expression, with an agonistic *α*-CLEC5A Ab, triggered in our hands significant transcriptomic changes in genes encoding proinflammatory cytokines; many of them belonging to the IL-1 family or being functional partners of IL-1. The involvement of the CLEC5A signaling in the activation of NLRP3 inflammasome and resulting IL-1b production have been already described during viral infections with DV and JEV [21] as well as in the infection with *L. monocytogenes* [10]. Very recently, Chen et al. reported the CLEC5A-associated activation of the NLRP3 inflammasome also in the context of an inflammatory disease, namely, in AOSD patients [26]. Importantly, our finding shows that an activation of the CLEC5A receptor even in the absence of infection or aseptic inflammation may activate the inflammasome and trigger the release of IL-1-like proinflammatory cytokines. Our study is to our knowledge the first report functionally connecting the selective CLEC5A activation (without any coexisting external or internal danger signals) with the activation of the inflammasome.

Interestingly, while CLEC5A activation in M0 (M-CSF) MdM led to significant transcriptomic changes, this was not the case when M1 (GM-CSF) MdM were exposed to the *α*-CLEC5A Ab.

Of note, the presence of strong proinflammatory maturation signals (IFN-g and LPS) in our *in vitro* M1 (GM-CSF) MdM differentiation protocol may have rendered these cells nonresponsive to the subsequent CLEC5A-mediated reprograming. Our observations on GM-CSF macrophages may thus correspond to the findings of Wortham et al. who demonstrated that the cytokine secretion from macrophages stimulated with an agonistic *α*-CLEC5A Ab combined either with LPS or with GM-CSF was much higher than in presence of the *α*-CLEC5A Ab alone. The addition of the *α*-CLEC5A Ab seemed to have rather a minor additive effect on the cytokine secretion in the above-mentionned study [14].

In addition to cytokines released from myeloid cells upon the CLEC5A receptor engagement, myeloid cells and T cells might interact in sterile conditions via an unknown CLEC5A-binding ligand. Joyce et al. previously proposed a model where galectin 9 (Gal9), a highly glycosylated galectin, could provide a functional bridge between myeloid cells and T cells [12]. However, in our hands, recombinant Gal9, in contrast to the *α*-CLEC5A Ab, failed to induce the CLEC5A-specific cytokine secretion from macrophages (data not shown); therefore, we did not study this hypothesis in more detail.

We aimed to understand whether the selective CLEC5A activation could support T cell priming in sterile conditions. First, we excluded a possibility that the CLEC5A-activating Ab may directly influence T cell activation. Second, we hypothesized whether activation of CLEC5A (known to function as a PAMP receptor during infection) could in consequence provide a potent maturation signal to antigen-presenting cells (APC), in analogy to other PAMP, e.g., TLR (Toll-like receptors), or to activating myeloid receptors like CD40 or CD80. If true, T cell activation might be in this scenario indirectly supported by the CLEC5A receptor activation on myeloid cells, for instance, via an enhanced expression of costimulatory molecules on APC together with a secretion of proinflammatory cytokines. In this case, CLEC5A could serve similarly to other myeloid cell receptors as a functional bridge between innate and adaptive immune cells. Interestingly, the exposure of CLEC5A-expressing M-CSF and GM-CSF MdM to the agonistic *α*-CLEC5A Ab did not enhance T cell response, while in some donors, the Ab even suggested the opposite effect, as shown by the significant decrease in T cell-derived IL-2 secretion and by significantly inhibited CD4+ T cell expansion. Of note, this effect could be observed only in the presence of the CLEC5A-expressing macrophages, while their replacement by supernatants harvested from MdM preincubated with the agonistic *α*-CLEC5A Ab did not reproduce the CLEC5A-mediated attenuation of T cell activation. Thus, we speculate that in our experimental conditions, soluble factors secreted from the CLEC5A-activated MdM are not sufficient to trigger the CLEC5A-mediated control of T cell proliferation. Instead, a yet unidentified cellular interaction between T cells and CLEC5A-expressing macrophages (e.g., via receptor-ligand binding) was required in our setting to exert the observed CLEC5A-dependent decrease in T cell activation. However, a more detailed study, involving more donors and conditions, could be necessary to elaborate more on this observation and on our hypothesis.

Altogether, the presence of the CLEC5A-activating Ab in the MdM+ T cell coculture did not support T cell activation, as it might have been suggested by the CLEC5A-mediated increase of proinflammatory cytokines and changes in myeloid cell phenotype. This finding may thus implicate that the activation of the CLEC5A receptor on myeloid cells in a noninfectious and noninflammatory context, i.e., in the absence of other proinflammatory stimuli, may not translate into a T cell-mediated immune response.

CLEC5A was previously described as an enhancer of the innate immune response, especially in the context of infection [9]. Similarly, TREM-1 receptor potentiates myeloid cell activation and proinflammatory cytokine release in response to TLR ligands or to other PAMPs [23]. In our hands, the selective CLEC5A activation led to an upregulation of TREM1 in a minority of donors (2/5) which may suggest rather a minor role of CLEC5A in the regulation of other myeloid checkpoints.

Interestingly, we observed that the CLEC5A agonist triggered significant changes in the expression of the MAFB transcription factor, reported previously to drive an anti-inflammatory (M2) phenotype of macrophages [22]. While CLEC5A-activated M0 (M-CSF) MdM downregulated the MAFB expression, the opposite trend was noted in case of M1 (GM-CSF) macrophages. In addition, we observed in our qRT-PCR study that the CLEC5A activation caused in GM-CSF macrophages a significant upregulation of the CLEC5A gene, which may suggest a self-perpetuating mechanism. Very recently, Shen et al. demonstrated that the overexpression of CLEC5A in M2 macrophages increased the level of M2 polarization [30]; thus, the upregulation of this gene in GM-CSF MdM may suggest a conversion of GM-CSF MdM towards a less proinflammatory phenotype. In terms of MdM interactions with other immune cells this effect may result in a failure to efficiently prime T cells.

Our observations may suggest that in sterile conditions the CLEC5A receptor might be responsible for a fine tuning in the process of macrophage activation. This effect could be achieved, for instance, by controlling the balance between the two opposite polarization scenarios of macrophages, in order to prevent the aberrant activation of M1 macrophages and the resulting unnecessary activation of T lymphocytes in the absence of pathogens.

CLEC5A has been suggested as a therapeutic target not only in the context of infection but also in inflammatory disorders (mostly based on the enhanced CLEC5A expression in these entities), for instance, in autoimmune-mediated skeletal disorders like rheumatoid arthritis or in systemic inflammatory response syndrome [3, 4]. Taking into account that CLEC5A orchestrates with other receptors on myeloid cells (TLR, C-lectins, and mannose receptor) and that its functional effects synergize with different stimuli and pathways, a further area of investigation remains open, i.e., whether a selective blockade of CLEC5A in aseptic conditions would be sufficient to achieve a therapeutic benefit.

## 5. Conclusions

We demonstrate here for the first time that CLEC5A receptor activation can trigger significant transcriptomic and phenotypic changes in human macrophages without the concomitant involvement of other receptors or coexisting danger signals. The elective CLEC5A agonist in sterile conditions led in our study to an ambiguous immune response, represented by a mixed pro- and anti-inflammatory cytokine response and by a modulation of myeloid cell receptors' expression in two opposite directions: upregulation of T cell activating markers (like CD80) together with immune suppressive M2-like markers (PD-L1, CD206). Functionally, the selective CLEC5A-mediated reprogramming of myeloid cells in aseptic conditions appeared insufficient to promote an autologous T cell activation and, in some donors, even slightly attenuated the adaptive T cell response. The CLEC5A receptor was suggested before to act as an immune amplifier, based on the studies conducted in the context of infection, inflammation or using ligands that concomitantly involve other receptors. In contrast, our work demonstrated that the selective activation of CLEC5A in the absence of additional stimuli can result in the immune regulation, as shown by the lack of productive T cell response. This way, the CLEC5A-mediated fine tuning of T lymphocyte function may represent an intrinsic protective immune mechanism against an undesired inflammation in aseptic conditions.

## Data Availability

All data to support the conclusions in the paper is provided within the manuscript and in the Supplementary material. The transcriptomic data set used to support the findings of this study is restricted to QC-curated data sets, while raw data may be available from the corresponding authors by request (due to commercial confidentiality).

## Abbreviations

Ab: Antibody
CLEC5A: C-type lectin-domain family 5 member A
CD: Cluster of differentiation
Ctrl: Control
DV: Dengue virus
ELISA: Enzyme-linked immunosorbent assay
FACS: Fluorescence activated cell sorting
GM-CSF: Granulocyte-macrophage colony-stimulating factor
IL: Interleukin
IP-10 (CXCL10): Interferon-gamma induced protein 10 kD
MCP-1 (CCL2): Monocyte chemoattractant protein-1
M-CSF: Macrophage colony-stimulating factor
MDC (CCL22): Macrophage-derived cytokine
MdM: Monocyte-derived macrophages
MFI: Median fluorescence intensity
MIP-1a (CCL3): Macrophage inflammatory protein-1 alpha
MIP-1b (CCL4): Macrophage inflammatory protein-1 beta
MMP: Matrix metalloproteinase
PAMP: Pathogen-associated molecular pattern
PBMC: Peripheral blood mononuclear cells
PBS: Phosphate buffering system
Syk: Spleen tyrosine kinase
SD: Standard deviation
TARC (CCL17): Thymus and activation-regulated chemokine
TLR: Toll-like receptor
TREM: Triggering receptor expressed on myeloid cells.

## Supplementary Materials

Supplementary Materials Supplementary Figures 1-6 and Supplementary Methods are available in a common PDF file.
