# Supplementary material for: Activation of the Innate Immune Checkpoint CLEC5A on Myeloid Cells in the Absence of Danger Signals Modulates Macrophages' Function but Does Not Trigger the Adaptive T Cell Immune Response
Source: J Immunol Res. 2022 Feb 25;2022:9926305. doi: 10.1155/2022/9926305 (PMC8896916; doi:10.1155/2022/9926305)
Supplement: Supplementary Materials — Supplementary Figures 1-6 and Supplementary Methods are available in a common PDF file. [file 9926305.f1.docx]

**Supplementary Figures**

**Supplementary Figure 1**

**Suppl. Fig. 1 Cytokine response of U937 WT cells to dengue virus (DV) lysates**

PMA differentiated U937 WT cells were treated with deactivated DV lysates at 0, 0.03, 0.3, 3, 30 and 100 µg/ml. Cell culture supernatant was collected after 2h, 8h, 16h, 24h, and 48h. MIP-1a, MIP-1b, TNF-a, IL-6, IL-10, IP-10, and IL-1b levels in supernatant were measured with Luminex.

**Supplementary Figure 2**

**A**

**B**

**Suppl. Fig. 2 CLEC5A expression on alveolar macrophages**

Alveolar macrophages isolated by adhesion from bronchioalveolar lavage fluid collected from three healthy donors aged 44 to 59 years were FACS stained with a PE-labelled a-CLEC5A Ab (grey line) or with isotype ctrl (grey shadow). CLEC5A expression is shown as an antibody vs isotype histogram overlay **(A)** or as median fluorescence intensity (MFI) of the CLEC5A Ab staining and isotype ctrl for each donor tested **(B)**.

Data represent three individual donors tested in one single measurement.**Supplementary Figure 3**

**A**

**B**

**Suppl. Fig. 3 CLEC5A agonism triggers transcriptomic changes in MdM**

mRNA expression of selected genes was determined by qRT-PCR in M-CSF/M0 **(A)** and in GM-CSF /M1 **(B)** macrophages incubated for 6h on 24 well plates coated either with 10 µg/ml a-CLEC5A Ab clone #283834 or with the corresponding isotype ctrl.

Results are indicated as the gene expression in samples treated with the a-CLEC5A Ab relative to samples treated with the isotype ctrl.

Values represent the mean and SD of three donors, *p<0.033, **p<0.002, ***p<0.001.

**Supplementary Figure 4**

**A**

**B**

**Suppl. Fig. 4 CLEC5A receptor agonism may influence the surface expression of TREM1 and TREM2 on MdM**

M0 MdM were incubated for 72h on culture plates coated either with a-CLEC5A Ab clone #283834 or with the corresponding isotype ctrl (10 µg/mL) and then FACS-stained with directly labelled monoclonal Abs against TREM1 (A) or TREM2 (B) surface receptors. Each marker is shown as median fluorescence intensity (MFI) calculated for each donor tested, while histograms illustrate an exemplary FACS fluorescence in one representative donor (grey line: MdM incubated with a-CLEC5A Ab; grey shadow: MdM with isotype ctrl).

Values represent the mean and SD from three technical triplicates per each donor. Shown are two independent experiments with n=6 donors. *p<0.033, **p<0.002, ***p<0.001.

**Supplementary Figure 5**

**Suppl. Fig. 5 A-CLEC5A Ab does not impact IL-2 secretion by T cells (when cultured alone)**

T cells purified from PBMC were activated with Dynabeads™ Human T-Activator CD3/CD28 kit on culture plates coated either with 10µg/mL a-CLEC5A Ab or with its corresponding isotype ctrl. IL-2 secretion in cell culture supernatants was measured after 48h by ELISA.

Values represent the mean and SD from three technical triplicates per donor. Shown is one experiment with three donors.

**Supplementary Figure 6**

**Suppl. Fig. 6 Supernatants from MdM pre-incubated with a-CLEC5A Ab do not influence T cell proliferation (as compared to isotype ctrl)**

PBMC labelled with a cell proliferation dye (CTV) were activated 4 days in presence of a-CD3 and a-CD28 Abs together with supernatants collected from M0 MdM pre-incubated either with a-CLEC5A Ab or isotype ctrl.

Shown is proliferation (defined as dilution of the cell proliferation dye) of CD3+ T cell population measured by FACS.

Values represent mean and SD from 3 individual PBMC donors.

**Supplementary Methods**

**Dengue virus (DV) - mediated cytokine secretion in U937 cells**

U937 wildtype cells were differentiated with PMA as described in the main section of the manuscript ‘Materials and Methods > Cytokine assay with U937 cells’. After differentiation, U937 cells were washed with medium and incubated with different concentrations (0 – 100 µg/ml) of inactivated DV lysate preparations (2BScientific, UK). After different time points (2– 48 h) cell culture supernatants were collected by centrifugation at 300g for 5min, transferred to 96 well Nunc PP V bottom plates (Thermo Fisher Scientific), and frozen at -20°C for later cytokine analysis. MIP-1α, MIP-1β, TNF-α, IL-6, IL-10, IP10 and IL1b levels were measured with Luminex.

**T cell assay**

Pan T cells were purified from frozen PBMC with the Pan T Cell Isolation Kit (Miltenyi Biotec) as described by the manufacturer. 1E5 Pan T cells were seeded in 96 well high binding plates (Corning) in RPMI 1640 (Gibco) supplemented with 5% Human Serum (Heat Inactivated from human male AB plasma, Sigma Aldrich). Plates were coated overnight at 4°C with 200 µL PBS solution containing either 10 µg/mLα-CLEC5A Ab clone #283834 or its corresponding isotype control clone #20116. Plates were washed twice with cold PBS before addition of the Pan T cells. Pan T cells were stimulated for 48h at 37°C with Dynabeads™ Human T-Activator CD3/CD28 (Gibco) as described by the manufacturer. IL-2 release in cell culture supernatants harvested after 48h was measured by ELISA as described by the manufacturer (R&D Systems).

**Quantitative real-time PCR (qRT-PCR)**

10^6^ MdM cells were seeded in a 24 well plate (Thermo Nunclo Delta #142475) coated with 10 µg of a-CLEC5A Ab or isotype control. Cells were incubated for 6h at 37°C before cells were lysed with RLT-buffer. Lysates were homogenized by use of QIAshredder (#79656, Qiagen). RNA isolation was performed by use of the King Fisher Flex and the MagMAX™-96 Total RNA Isolation Kit (AM1830) as described by the manufacturer. RNA concentration was determined with nanodrop. For qRT PCR the iTaq™Universal Probes One-Step Kit (#172-5140, Bio-rad) was used. For each reaction 200 ng RNA were mixed with 20x TaqMan Gene Expression Assay (ThermoFisher Scientific) and the above-mentioned kit components as described by the manufacturer. Samples were analyzed by QuantStudio 7 (Life Technologies). Fold changes of the different transcripts were calculated (2-ΔΔCt method) and normalized to the house-keeping gene ß-actin.

The following 20x TaqMan gene Expression kits from Thermo Fisher Scientific were used: MAFB (Hs00534343_s1), MERTK (Hs00179024_m1), CD14 (Hs02621496_s1), CD163 (Hs00174705_m1), CD206 (MRC-1, Hs00267207_m1), CD209 (DC-Sign, Hs00253550_m1), FLT1 (Hs01052961_m1), CD274 (PD-L1, Hs01125301_m1), Il1RAP (Hs00895050_m1), CLEC5A [Hs04398399_m1) and β-Actin (Hs01064292_g1).

**PBMC assay with macrophage supernatants**

Supernatants were harvested from M0/ M-CSF MdM (generated from PBMC as described in Materials and Methods) that were cultured 72h at 5x10^4^ cells in 100 µl/well on 96-well plates pre-coated overnight either with a-CLEC5A Ab or with isotype ctrl (10 µg/ml).

Next, PBMC from 3 different donors were labelled with 5 µM CellTrace™ Violet Cell Proliferation Kit (Thermo Fisher Scientific) according to the manufacturer’s instruction and plated at 2x10^5^ cells in 100 µl/well on a 96-well plate pre-coated with 1.25 µg/ml a-CD3 (OKT3) Ab in presence of 0.5 mg/ml soluble a-CD28 Ab and 100 µl/well macrophage supernatants (pre:diluted 1:2).

After 4 days PBMC were harvested, stained with a-CD3 Ab and cell proliferation (defined as CTV dilution in CD3+ cells) was measured by FACS.
